# Pedigree analysis and estimates of effective breeding size characterize sea lamprey reproductive biology

**DOI:** 10.1111/eva.13364

**Published:** 2022-03-15

**Authors:** Ellen M. Weise, Kim T. Scribner, Jean V. Adams, Olivia Boeberitz, Aaron K. Jubar, Gale Bravener, Nicholas S. Johnson, John D. Robinson

**Affiliations:** ^1^ 3078 Department of Fisheries and Wildlife Michigan State University East Lansing Michigan USA; ^2^ 3078 Department of Integrative Biology Michigan State University East Lansing Michigan USA; ^3^ US Geological Survey ‐ Great Lakes Science Center Ann Arbor Michigan USA; ^4^ USFWS Ludington Biological Station Manistee Michigan USA; ^5^ Fisheries and Oceans Canada Sea Lamprey Control Centre Sault Ste. Marie Ontario Canada; ^6^ US Geological Survey Great Lakes Science Center Hammond Bay Biological Station Millersburg Michigan USA

**Keywords:** effective breeding number, genetic assessment, mixture models, pedigree analysis, *Petromyzon marinus*, RAD‐Capture, SNPs

## Abstract

The sea lamprey (*Petromyzon marinus*) is an invasive species in the Great Lakes and the focus of a large control and assessment program. Current assessment methods provide information on the census size of spawning adult sea lamprey in a small number of streams, but information characterizing reproductive success of spawning adults is rarely available. We used RAD‐capture sequencing to genotype single nucleotide polymorphism (SNP) loci for ~1600 sea lamprey larvae collected from three streams in northern Michigan (Black Mallard, Pigeon, and Ocqueoc Rivers). Larval genotypes were used to reconstruct family pedigrees, which were combined with Gaussian mixture analyses to identify larval age classes for estimation of spawning population size. Two complementary estimates of effective breeding size (*N*_b_), as well as the extrapolated minimum number of spawners (*N*_s_), were also generated for each cohort. Reconstructed pedigrees highlighted inaccuracies of cohort assignments from traditionally used mixture analyses. However, combining genotype‐based pedigree information with length‐at‐age assignment of cohort membership greatly improved cohort identification accuracy. Population estimates across all three streams sampled in this study indicate a small number of successfully spawning adults when barriers were in operation, implying that barriers limited adult spawning numbers but were not completely effective at blocking access to spawning habitats. Thus, the large numbers of larvae present in sampled systems were a poor indicator of spawning adult abundance. Overall, pedigree‐based *N*_b_ and *N*_s_ estimates provide a promising and rapid assessment tool for sea lamprey and other species.

## 1 INTRODUCTION

Invasive species are a substantial threat to biodiversity, and management intervention is often required to mitigate their effects on the ecosystem. Annual control programs to reduce the population size of widespread invasive species (Prior et al., 2018) often include strategies to reduce recruitment and spread, like barriers that limit access to spawning habitat (Sharov & Liebhold, 1998). More recently, genetic control techniques like the release of sterile individuals or gene drive have been developed as additional options for control (Bajer et al., 2019).

Genetic technologies, used in combination with field techniques, allow managers opportunities to efficiently and cost‐effectively sample large areas to quantify the presence of species, community composition, and species biomass and abundance. Environmental DNA was used as an early detection tool for specific invasive species like American bullfrogs (*Lithobates catesbeianus*) and invasive shellfish species, allowing for rapid response after the invasion (Dejean et al., 2012; Leblanc et al., 2020). To evaluate widespread invasions, demographic modeling has been used to track the spread of invasive species across a system to determine the introduction point and generate hypotheses for the mechanism of introduction (Blakeslee et al., 2017; Sherpa et al., 2019). Additionally, determining the founding effective size of an invasive population can provide insight into the mechanism of invasion and the severity of the bottleneck present in an introduced species (Nathan et al., 2015; Sard et al., 2019). Genetic parentage assessment and effective size estimates can be used to evaluate the size and diversity of spawning populations as an annual assessment tool for managed or invasive populations, although this type of application is less common than applications for conserved populations (Levine et al., 2019; Taylor et al., 2021). This tool can be used to evaluate the success of control efforts for an invasive species.

Sea lampreys (*Petromyzon marinus*) are a widespread invasive species in the Laurentian Great Lakes (McGeoch et al., 2010). The expansion of the Welland Canal in 1919 allowed sea lamprey to spread from Lake Ontario to the rest of the Great Lakes by 1938 (Lawrie, 1970). Sea lamprey contributed to major declines in commercially valuable fish species like lake trout (*Salvelinus namaycush*) and lake whitefish (*Coregonus clupeaformis*) throughout the Great Lakes basin (Heinrich et al., 2003; Koonce et al., 1993; Lawrie, 1970). As a result of the ecological and economic impacts of the invasion, an annual control and assessment program was implemented in the 1950s to reduce sea lamprey abundance and assist recovery of native fish populations (Smith & Tibbles, 1980).

The primary methods of sea lamprey control since the 1950s have been physical barriers that block adults from reaching spawning habitat and application of the selective lampricide 3‐trifluormethlyl‐4‐nitrophenol (TFM) to kill larvae (Applegate, 1950; McDonald & Kolar, 2007; Smith & Tibbles, 1980). Several barrier designs have been implemented since the beginning of the control program to reduce migration of sea lamprey into streams (Lavis et al., 2003; McLaughlin et al., 2007). However, these barriers also impede the movement of numerous ecologically and culturally important native fish species (Jensen & Jones, 2018). Adjustments and alternative barrier designs have been used to reduce effects on native fish (Katopodis et al., 2009), such as seasonal electric barriers or the addition of a fish ladder (Lavis et al., 2003; Zielinski et al., 2019). Many barriers have been removed altogether, resulting in an increase in spawning habitat for sea lamprey throughout the Great Lakes. Additionally, sea lamprey larvae are occasionally found upstream in systems with barriers. In these cases, managers want to know when and how many adult sea lampreys escaped upstream of the barrier. However, given uncertainty in stock‐recruitment relationships and a limited ability to age larvae, these questions are largely unanswered (Dawson et al., 2009; Jones, 2007). Population genetic data can address these questions by estimating the number of successfully spawning adults that contributed to a year class of larvae and tracking the movements of individuals from each year class over several years (Ovenden et al., 2016; Sard et al., 2020).

Sea lampreys are semelparous and have a multistage anadromous life history that can span up to 9 years (Applegate, 1950). Adults migrate upstream, spawn in spring and summer, and die afterward (Johnson et al., 2015). Larvae reside in streams and lentic areas near streams and feed on algae and detritus while burrowed into soft sediment (Dawson et al., 2015). After two (Morkert et al., 1998) to seven years (Manion & Smith, 1978) in the larval stage, larvae undergo metamorphosis, migrate to the Great Lakes, and feed on fishes for 12–18 months. Adult sea lampreys do not return to natal streams to spawn (Bergstedt & Seelye, 1995), but instead, stream selection is guided by chemosensory cues released by larval sea lamprey (Fissette et al., 2021). Therefore, population structure of sea lamprey is weak relative to homing fishes (Bryan et al., 2005). Key uncertainties regarding sea lamprey demographics include stock‐recruitment relationships (Dawson & Jones, 2009), larval survival (Jones et al., 2009), and age at metamorphosis (Griffiths et al., 2001; Treble et al., 2008), in part, because of difficulty aging larvae (Dawson et al., 2021).

Recent developments in sequencing technologies, the declining costs of high‐throughput sequencing, and expanding genomic resources for sea lamprey (Sard et al., 2020; Smith et al., 2013, 2018) present an opportunity to incorporate population genomic methods and data analysis into invasive species assessment efforts. Reduced representation sequencing technologies such as restriction‐site associated DNA (RAD) sequencing (Baird et al., 2008) and locus‐targeted RAD‐Capture (Ali et al., 2016) allow for the collection of genome‐scale data from large population‐level sample sizes. The use of genomic data to study invasive species populations offer numerous applications to assist managers in assessing sea lamprey reproductive ecology in natural stream settings. These data also provide a means to evaluate the effectiveness of experimental barriers and gain additional insight into sea lamprey reproductive ecology in Great Lakes tributaries.

Several parameters are routinely estimated based on genetic data to quantify spawning adult abundance and reproductive success (e.g., Sard et al., 2020 for sea lamprey). Effective population size (*N*_e_) is the size of an idealized population that experiences the same amount of genetic drift, inbreeding, or loss of diversity as the population in question (Wright, 1931). *N*_e_ has been used in assessments of populations and as an indicator of potential for future declines in abundance (Antao et al., 2011). Low *N*_e_ can also be an indicator of low levels of genetic diversity in a population (Frankham, 2010). In many species, individuals from multiple age classes produce offspring simultaneously, resulting in overlapping generations (Waples et al., 2014). In this situation, the effective number of breeding individuals contributing to a spawning event (*N*_b_) can also be estimated using samples from a single year class (Robinson & Moyer, 2013; Waples et al., 2014; Waples & Do, 2010).

*N*_e_ can be reduced relative to census size by several factors, including skewed sex ratios and variation in reproductive success (Waples, 2010). The ratio of *N*_b_ to *N*_e_ has been shown to be strongly associated with life history traits such as time to sexual maturity and adult lifespan (Waples et al., 2013). In addition to *N*_b_, the minimum number of spawning adults (*N*_s_) can also be calculated from reconstructed pedigrees as the minimum number of parental genotypes required to produce the sampled offspring genotypes. Using approaches to estimate total species richness from the field of community ecology (Chao, 1987; Heltshe & Forrester, 2009), information on the contribution of inferred parental genotypes to sampled larvae can provide estimates of the total number of parents contributing to a cohort (Hunter et al., 2020), including asymptotic estimates of total spawning adult numbers (Sard et al., 2021).

*N*_b_ can be estimated from population genetic or genomic data using several methods. Here, we apply two computationally different approaches to demonstrate consistencies in estimates of sea lamprey effective breeding size: linkage disequilibrium (LD; Waples & Do, 2010) and sibship frequency (SF; Wang, 2009). The LD method uses nonrandom associations of alleles across loci that result from finite population size or physical linkage (Hill, 1981a,1981b). If chromosomal locations of loci can be established and effects of physical linkage can be removed, LD resulting from finite breeding population size can be estimated to characterize effective breeding size (Waples et al., 2016). In contrast, SF uses the frequency of sibling relationships identified in a reconstructed pedigree (Wang, 2009), where sampled offspring are used to reconstruct unsampled parental genotypes (Bravington et al., 2016; De Barba et al., 2010; Keogh et al., 2007).

In this study, our objective was to estimate effective breeding size and minimum number of spawners for larval sea lamprey cohorts collected from streams above barriers to upstream migration in three locations in the northern Lower Peninsula of Michigan: the Black Mallard, Pigeon, and Ocqueoc Rivers. In all three locations, the presence of larvae upstream of barrier locations raised concerns about barrier failure to impede spawning migrations. We used the estimates above to evaluate barrier efficacy in all three systems. Furthermore, we used reconstructed pedigrees of each collection along with Gaussian mixture analysis to estimate the number of larval age classes present in each system. We discuss possible explanations for barrier failure in these systems, highlight the utility of population genomic data for rapid assessment of spawning populations, and describe how genetic data can be integrated into monitoring and control efforts for invasive species.

## 2 METHODS

### 2.1 Study system and sample collection

**FIGURE 1:**
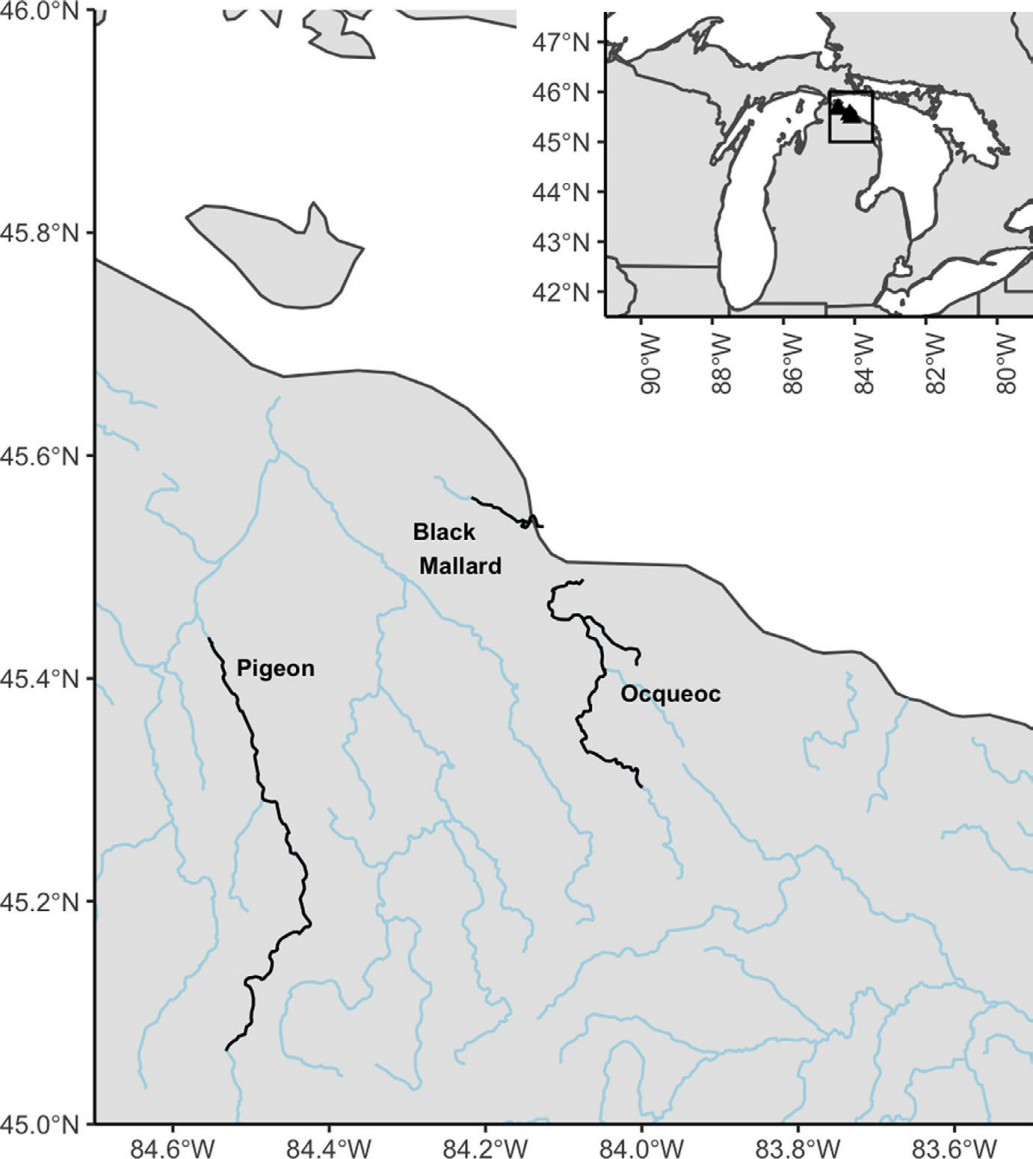
Map of the study area where larval sea lamprey was collected. The Black Mallard River is separated into upper and lower sections by Black Mallard Lake. The top‐right inset shows the location of the sampled river systems in the Great Lakes region. River lines in black denote sampling locations of the river systems; blue lines denote all other rivers in the region

Sampling of larval sea lamprey was conducted in the Black Mallard, Ocqueoc, and Pigeon Rivers, which are located in the northern Lower Peninsula of Michigan, USA (Figure 1). In all three systems, larval sea lampreys were collected above barriers designed to preclude access to spawning habitat. The spatial extent of sampling was extensive in all rivers to define the distribution of the larval sea lamprey infestations and to obtain a comprehensive spatial representation of larvae produced from all family groups.

The Black Mallard River had an electric barrier installed in 2016 following a lampricide treatment that occurred in June 2015. In September 2017, larvae in the section of the Black Mallard River downstream from Black Mallard Lake were collected using backpack electrofishing (*n* = 387). Sea lampreys were sampled from habitat spanning 500‐m upstream and downstream of Ocqueoc Lake Road and U.S. Highway 23. These two sampling points represented the furthest upstream and downstream extent of the lower river with stream substrate suitable for larval sea lamprey and covered about 50% of the available larval habitat in the lower river. Lampricide treatment of the Black Mallard River downstream of Black Mallard Lake occurred in July 2018, and dead sea lamprey larvae were collected post‐treatment by two staff that walked the entire stream length from Ocqueoc Lake Road to U.S. 23 (*n* = 667). These collections will be referred to hereafter as the “Lower Black Mallard River.” Variation in larval length in the samples raised concerns that larvae might include individuals from multiple age classes that would indicate that the barrier had failed repeatedly. Larvae were also collected upstream of Black Mallard Lake in May 2019 when lampricide was applied. Two staff walked 2‐km downstream and 2‐km upstream from Elah Road and covered the entire known distribution of larval sea lamprey in the upper river. Surveys were also conducted upstream and downstream of Elah Road post‐lampricide treatment, but no sea lampreys were found. This collection will be referred to hereafter as the “Upper Black Mallard River.”

The Ocqueoc River has had an electric barrier in place since 1951 (Smith & Tibbles, 1980), with a permanent barrier installed since 1999. The area upstream of the barrier is the site of annual experiments that involve the release of thousands of adult female sea lamprey (Buchinger et al., 2020; Johnson et al., 2014; Wagner et al., 2018). Adult males are not included in experimental releases, so no successful spawning was expected in the system. However, a population of larvae was found above the barrier in 2018, and surveys conducted throughout the river identified a roughly 5‐km infested reach downstream of Ocqueoc Falls. Lampricide was subsequently applied in the stream in September 2018, and larvae were collected during treatment using dip nets and drift nets by four staff that walked the entire infested area (*n* = 389). Surveys for dead sea lamprey were also conducted at Pomranke Road (5‐km downstream of infested area) and in Silver Creek (tributary to Ocqueoc River), but no sea lampreys were found.

The Cheboygan River system has a dam at the mouth of the river but has small sea lamprey populations, which complete the juvenile parasitic phase of their life cycle in several upstream lake and stream systems; the Pigeon River is one such tributary (Johnson et al., 2020). To depress or eradicate these populations, releases of sterile males have been used as a supplemental control technique to limit successful female reproduction (Johnson et al., 2020; Kaye et al., 2003; Twohey, 2016). During these efforts, a small number of larvae (*n* = 29) were found at Webb Road in the Pigeon River in September 2018. Ten other locations spanning a 55‐km section of the Pigeon River were also sampled in 2018 (some upstream and some downstream), but no sea lampreys were collected at those other sites.

Sea lamprey collected from all systems were euthanized, preserved in 95% ethanol, and returned to the laboratory (IACUC ID: PROTO201800143). Length and weight were measured for each individual sampled, to estimate age class. A tissue sample was taken for genetic analysis.

### 2.2 RAD‐capture Sequencing

DNA was extracted from each larva using DNeasy blood and tissue kits (QIAGEN, Carlsbad, CA). DNA concentrations were initially quantified using a Nanodrop ND‐1000 Spectrophotometer (ThermoFisher Scientific, Waltham, Massachusetts) and Quant‐iT^TM^ PicoGreen^TM^ dsDNA Assay Kits (ThermoFisher Scientific, Waltham, Massachusetts) on a QuantStudio 6 Flex Real‐Time polymerase chain reaction (PCR) system (Thermo Fisher Scientific Inc., Waltham, Massachusetts). Samples were standardized to a concentration of 10 ng/µl for RAD sequencing.

RAD library preparation was performed on 100 ng of DNA per individual using a modified version of the BestRAD protocol (Ali et al., 2016). DNA was digested using an *SbfI* restriction enzyme, and a biotinylated BestRAD adaptor was ligated to the DNA, which functioned as an individual barcode. DNA from groups of 96 barcoded individuals was pooled, concentrated, and sheared using a Covaris m220 focused‐ultrasonicator (Covaris, Woburn, Massachusetts) using manufacturer recommended settings for a fragment size of 325 bp. Next, a streptavidin bead binding assay was used to select DNA fragments with RAD tags attached, and a size selection was used to select only the target size fragments for sequencing. Size selection was done using Ampure beads with a 22:50 ratio to select long fragments and a 13:72 ratio to separate target size fragments from shorter fragments. Finally, NEBNext Kits (New England BioLabs Inc, Ipswich, Massachusetts) were used to ligate plate‐specific Illumina adaptors and a universal adaptor for sequencing.

Library concentrations were quantified using a Picogreen assay, and the quality of the library was assessed via Tapestation (Agilent, Santa Clara, California) analysis. Libraries were pooled in groups of four to be enriched for a set of 3446 RAD loci that are known to be variable in sea lamprey populations (Sard et al., 2020). Loci were targeted using the RAD‐capture approach (Ali et al., 2016) with a custom MyBaits hybridization capture kit (Arbor Biosciences, Ann Arbor, MI) following the manufacturer recommended protocol. Eleven cycles were used in the final amplification step in the capture kit. Libraries were sequenced on four Illumina HighSeq X lanes at Novogene (Chula Vista, CA) using paired‐end 150 base‐pair sequencing. Sequencing data for the project are available on the NCBI sequence read archive (Accession Number: PRJNA763927).

### 2.3 Genotyping analysis

Raw sequence data were processed using a bioinformatic pipeline described in Sard et al. (2020). Prior to the pipeline, a quality control report was constructed for each library using FastQC (Andrews, 2010) and evaluated. First, sequences from the HighSeq X run were oriented using the custom perl function bRAD_flip_trim.pl (originally developed by Paul Hohenlohe, University of Idaho, and modified by Brian Hand and Seth Smith, University of Montana) and demultiplexed using the Stacks 2.0 (Catchen et al., 2013) module “process_radtags.” PCR duplicates were removed using “clone_filter.” Next, sequences were quality trimmed using trimmomatic with a minimum length of 50, a sliding window of 4 bases, and a minimum quality score of 15 (Bolger et al., 2014). Sequences were then mapped to the sea lamprey reference genome (Smith et al., 2018), and indexed using bwa and bwa‐mem (Li, 2013; Li & Durbin, 2010). Samtools (version 1.9) was used to sort reads with default settings (Li et al., 2009). Genotypes were called using the Stacks 2.4 (Catchen et al., 2013) module “gstacks,” and the module “populations” was used to generate a.vcf file containing genotypes for all individuals. To avoid the inclusion of paralagous loci in the data set, the software HDplot (McKinney et al., 2017) was used to identify and exclude potential paralogs. Loci were removed if observed heterozygosity was >0.6, or the read ratio deviation statistic (D; McKinney et al., 2017) in heterozygotes was greater than 7 in absolute magnitude. Individuals with more than 80% missing SNPs in the set were removed from analysis to minimize missing data. Each SNP set was checked for significant deviance from Hardy–Weinberg equilibrium across populations using the output from the Stacks 2.0 “populations” function prior to use in downstream analyses. Final genotype calls were filtered to exclude samples with <8X coverage. To determine which SNPs were located on the sections of the genome targeted by the RAD‐capture baits, the position of each SNP was compared to the genome position ranges for each RAD‐capture tag (Sard et al., 2020).

To ensure that all individuals were sea lamprey samples rather than misidentified native Northern or American brook lamprey (*Ichthyomyzon fosso*; *Lampetra appendix*), comparative analyses were conducted. RAD‐capture sequences of known American and Northern brook lamprey (*n* = 10) were aligned to the sea lamprey genome along with sampled individuals. A principal component analysis (PCA) was conducted for both native lamprey species and sampled individuals to identify clusters of individuals based on genotypes. All sampled individuals were compared to look for individuals that were identified as sea lamprey but clustered with native species, and none were found (Figure S1a‐c). Additionally, neighbor‐joining phylogenetic trees were constructed using SNP differences as an additional check for misidentified individuals. All trees separated along species lines with no sampled individuals sorted with either native lamprey species.

### 2.4 Gaussian mixture analyses

**FIGURE 2:**
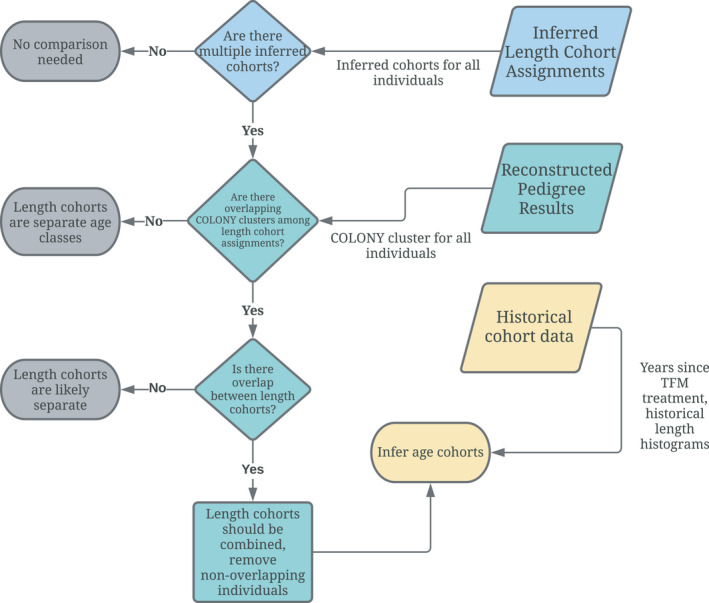
A flow chart describing how inferred cohort assignments from the Gaussian mixture models are combined with information in the reconstructed pedigrees

Offspring from sea lamprey and other fish species often exist in mixtures of individuals of different ages (cohorts), and these age classes need to be separated for estimation of *N*_b_ and *N*_s_. We developed a novel extension of Gaussian mixture methods by combining mixture models with reconstructed pedigrees (Figure 2). Given the semelparous life history of sea lamprey, full‐ and half‐sibling relationships should not span different cohorts; therefore, all individuals connected in the pedigree were assumed to be from the same age class. Aging methods like statolith aging have been found to be unreliable (Dawson et al., 2015), and length‐based aging methods have been primarily used by management agencies for sea lamprey (Hardisty & Potter, 1971; Sethi et al., 2017; Slade et al., 2003). Lengths of sea lamprey larvae were used in Gaussian mixture analyses to classify individuals into putative age classes prior to estimation of effective breeding size (*N*_b_) and the minimum number of spawners (*N*_s_). Mixture analyses were conducted separately for each stream and each collection year due to variation in larval length between streams and collection years.

Mixture models were constructed using the R packages BayesMix (Grün & Leisch, 2010) and bmixture (Mohammadi et al., 2013) to infer the number of age classes (K) and generate individual assignments to those cohorts. We used two different approaches to assess the number of cohorts represented by a sample of sea lamprey larvae. Birth‐death Marcov chain Monte Carlo (MCMC) treats K as a model parameter that is allowed to increase or decrease in successive steps of the MCMC chain to provide posterior probabilities for each potential K value (Mohammadi et al., 2013; Stephens, 2009). Rousseau and Mengersen (2011) proposed a cluster‐determining method that involves fitting a mixture model with a large K value and eliminating clusters with membership proportions below a certain cutoff (between 0.01 and 0.05; Nasserinejad et al., 2017). For this project, a cutoff of 0.035 and a K of 10 were used. The consensus from birth‐death MCMC and the Rousseau and Mengersen (2011) approaches was used as the K value in a BayesMix model to determine individual assignments to clusters. If consensus was not reached, the output with a higher posterior probability was used as the K value. All analyses were conducted in R (version 3.6.2). All scripts, data, and documentation for these analyses are available at https://github.com/weiseell/NbdLamprey.

### 2.5 Reconstructed pedigrees

SNP genotype data were used to reconstruct pedigrees for larvae sampled from all locations. SNP loci were selected from the filtered group of SNPs for each population using the following criteria: minimum separation of adjacent SNP loci of 1MB to reduce the influences of physical linkage, variant position with the highest minor allele frequency (MAF ≥0.05), and highest percent of individuals genotyped (with minimum criteria of 80%). If two or more SNPs met all three criteria equally, a random SNP was selected from that group. For each stream system, pedigree analysis was conducted in Colony version 2.0.6.6 (Jones & Wang, 2010) using the full‐likelihood approach. Due to differences in sample size among systems, a medium length run was used for the Black Mallard and Ocqueoc Rivers, and a long run was used for the Pigeon River. Other input parameters included unknown allele frequencies, polygamous mating, no sibship scaling, or prior sibship were reported, and the genotyping error rate was set at 0.001. All other parameters were kept at default settings.

Colony clusters from the reconstructed pedigree were compared to cohorts determined by the Gaussian mixture analysis to check for discrepancies between clusters of related individuals in the pedigree and cohorts assigned by the mixture analysis. A family cluster from Colony is defined as a group of offspring that are connected in the pedigree through parentage but are not necessarily full‐ or half‐siblings. For example, if offspring 1 and offspring 2 are half‐siblings, and offspring 2 and offspring 3 are half‐siblings, then offspring 1 and offspring 3 are considered to be in the same Colony cluster due to their connection in the pedigree through offspring 2. For each collection with multiple inferred cohorts from the Gaussian mixture analysis, individuals were evaluated for the level of family overlap between inferred cohorts. If there was no overlap of Colony cluster groups between inferred cohorts, they were left separate for subsequent analysis. If individuals in the inferred cohorts were related (as full‐ or half‐siblings), these individuals were combined into a single cohort for subsequent analyses. If there were multiple sample collections from the same location, the comparison was repeated to determine which cohorts should be combined across collections and to approximate growth between collections to help separate year classes. Length histograms from previous studies (Dawson et al., 2009) were used as a benchmark for estimating the age classes associated with each identified length cohort, and information on barrier installation and TFM treatment years were used to limit the number of potential age clusters in analyses used to estimate K from each sample. A flowchart of the decision‐making process is shown in Figure 2. To assess the sensitivity of our results, the process was repeated with full‐sibling groups, which produced the same results as the analysis done with Colony cluster groups.

### 2.6 *N*_b_ and *N*_s_ estimates

Colony was used to estimate *N*_b_ using the SF method (Wang & Santure, 2009), and mean ($\bar{k}$) and variance (*V*_k_) of adult reproductive success (number of offspring assigned based on the pedigree produced from the full‐likelihood implementation in Colony) were calculated for the contributing individuals in the reconstructed parental populations. *N*_s_ was generated using the number of inferred parents represented in each cohort. *N*_s_ was extrapolated using a “parentage accumulation curve,” which is akin to a species accumulation curve (Colwell et al., 2004; Israel & May, 2010; Rawding et al., 2014), to count the number of distinct parental genotypes as the number of offspring genotyped in the sample increases (Hunter, 2018; Sard et al., 2021). Briefly, the specaccum function from the R package vegan (Oksanen et al., 2019) was used to generate pedigree accumulation curves, and the total number of parental genotypes contributing to each cohort (${\hat{N}}_{s}$) was estimated using the Chao (Chao, 1987) and jackknife (Heltshe & Forrester, 2009) methods in the vegan function specpool (Oksanen et al., 2019).

The SNP panel used for estimates of *N*_b_ from the LD method (LD) was selected with a separate set of criteria due to inherent differences in the estimation methods. SNPs were selected to only include loci in regions of the genome targeted by the Sard et al. (2020) Rapture panel. Within those RAD tags, SNPs with the highest percentage of individuals genotyped were selected, and ties were broken with a random variable. NeEstimator (Do et al., 2013) was used for each cohort and stream sample with only the LD method selected, no comparisons within chromosomes were allowed (to avoid LD due to physical linkage of SNP markers; Waples et al., 2016). SNPs with a MAF <0.05 were removed to avoid potential upward bias in the *N*_b_ estimates from low‐frequency alleles (Waples & Do, 2010). Estimates were generated using an allele frequency inclusion criterion of p_crit_ = 0.05, and jackknife confidence intervals produced by NeEstimator were used (Jones et al., 2016). All analyses for *N*_b_, *N*_s_, and ${\hat{N}}_{s}$, with the exception of the Colony and NeEstimator programs, were conducted in R (version 3.6.2; R Core Team, 2019), and all scripts and documentation for these analyses are available at https://github.com/weiseell/NbdLamprey.

## 3 RESULTS

### 3.1 Genotyping analysis

Sequencing generated more than 3 billion total reads with an average of approximately 2 million reads for each individual (range: ~2000–12 million reads). After removal of PCR duplicates and quality filtering, reads were mapped to the sea lamprey reference genome (Smith et al., 2018). Of the filtered mapped reads, 88% were from sections of the genome targeted by the Rapture panel developed by Sard et al. (2020). Average sequencing depth in targeted regions was 34X. The SNPs targeted by the Rapture panel also had a higher proportion of loci with MAF >0.05 (0.25) when compared to nontargeted SNPs (0.177), and a higher mean proportion of individuals genotyped per SNP (on‐target = 0.56, off‐target = 0.20).

### 3.2 Mixture analyses and reconstructed pedigrees

**FIGURE 3:**
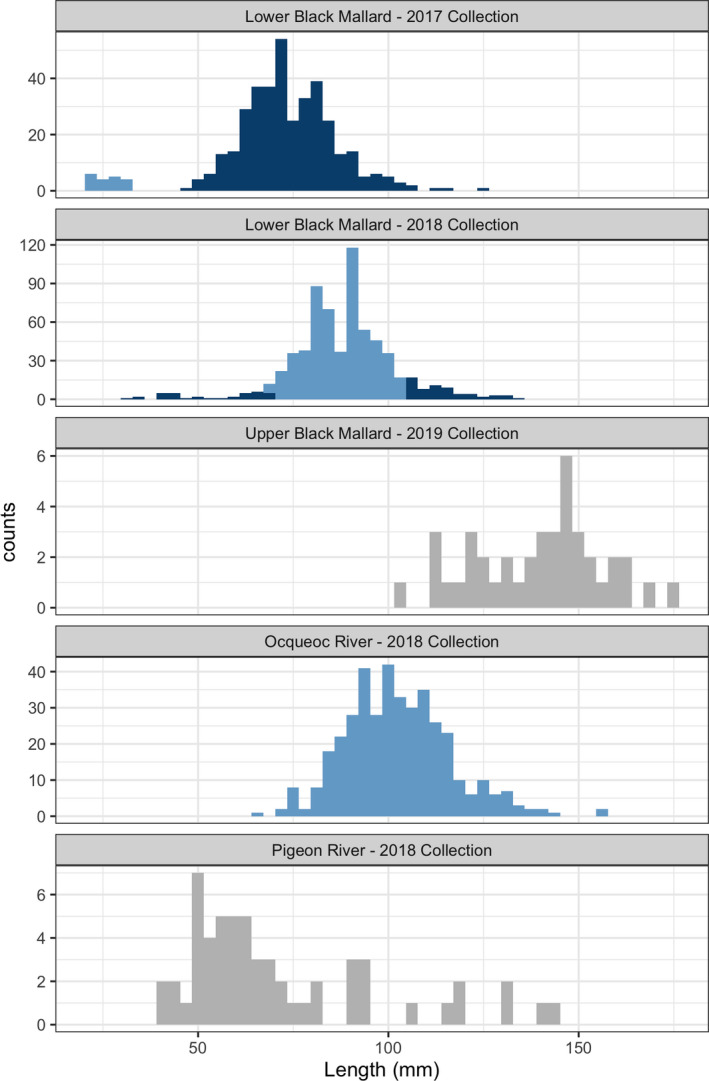
Length frequency distributions for larval sea lamprey from all rivers and collection years fill colors represent individual cluster assignment from the Gaussian mixture analysis. If mixture models were not completed due to small sample size, length histograms are included and shaded as a single cohort

**FIGURE 4:**
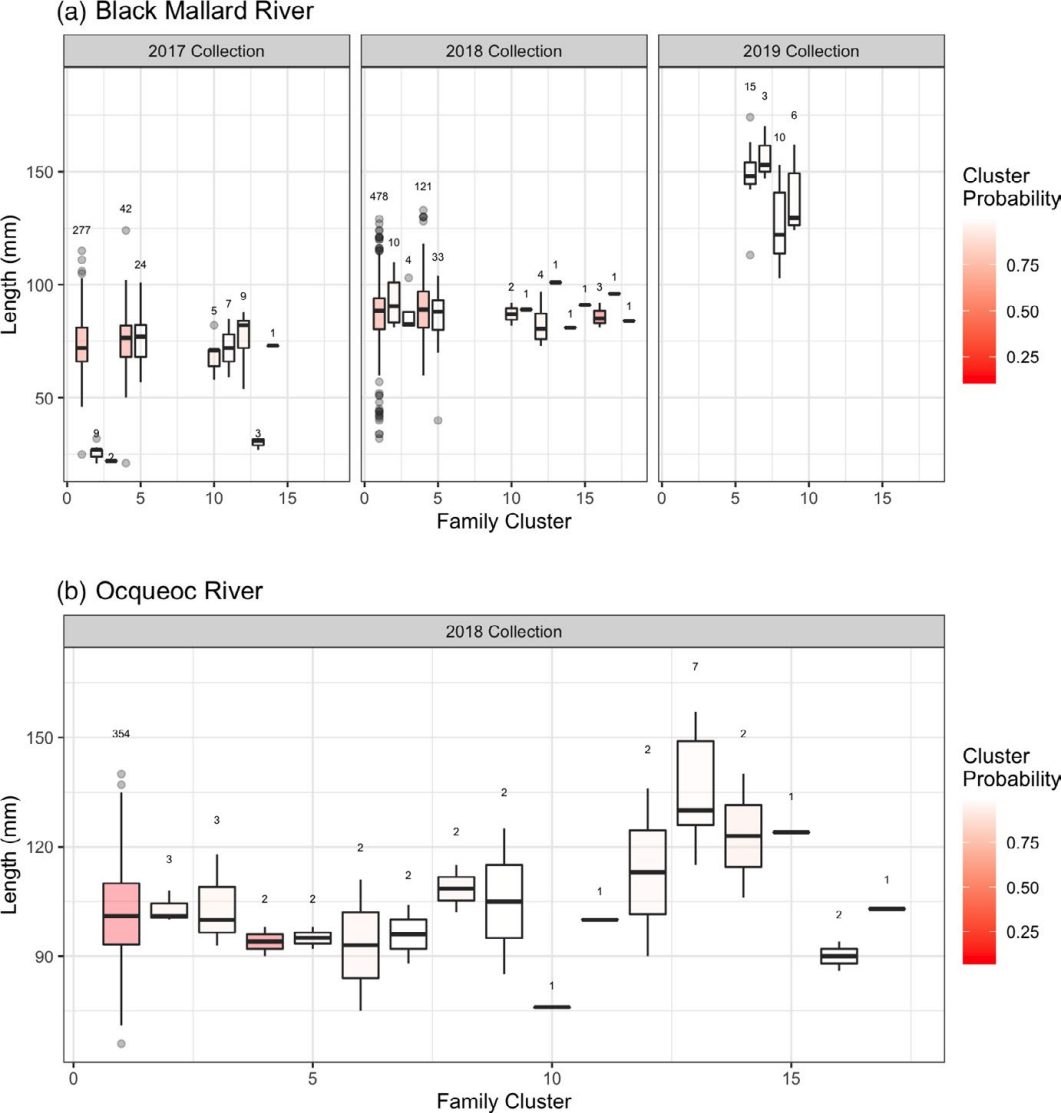
Boxplots of length distributions for each sea lamprey Colony cluster from the Lower Black Mallard River (a) and the Ocqueoc River (b). Colony clusters are defined as groups of offspring in the pedigree that are connected by parentage but are not necessarily full‐ or half‐siblings. Plots are separated by collection. The probability that the Colony cluster cannot be split is represented by a continuous shading scale for both subplots (red clusters have a lower probability, white clusters have a higher probability). The number above each boxplot refers to the number of individuals represented in the cluster

**FIGURE 5:**
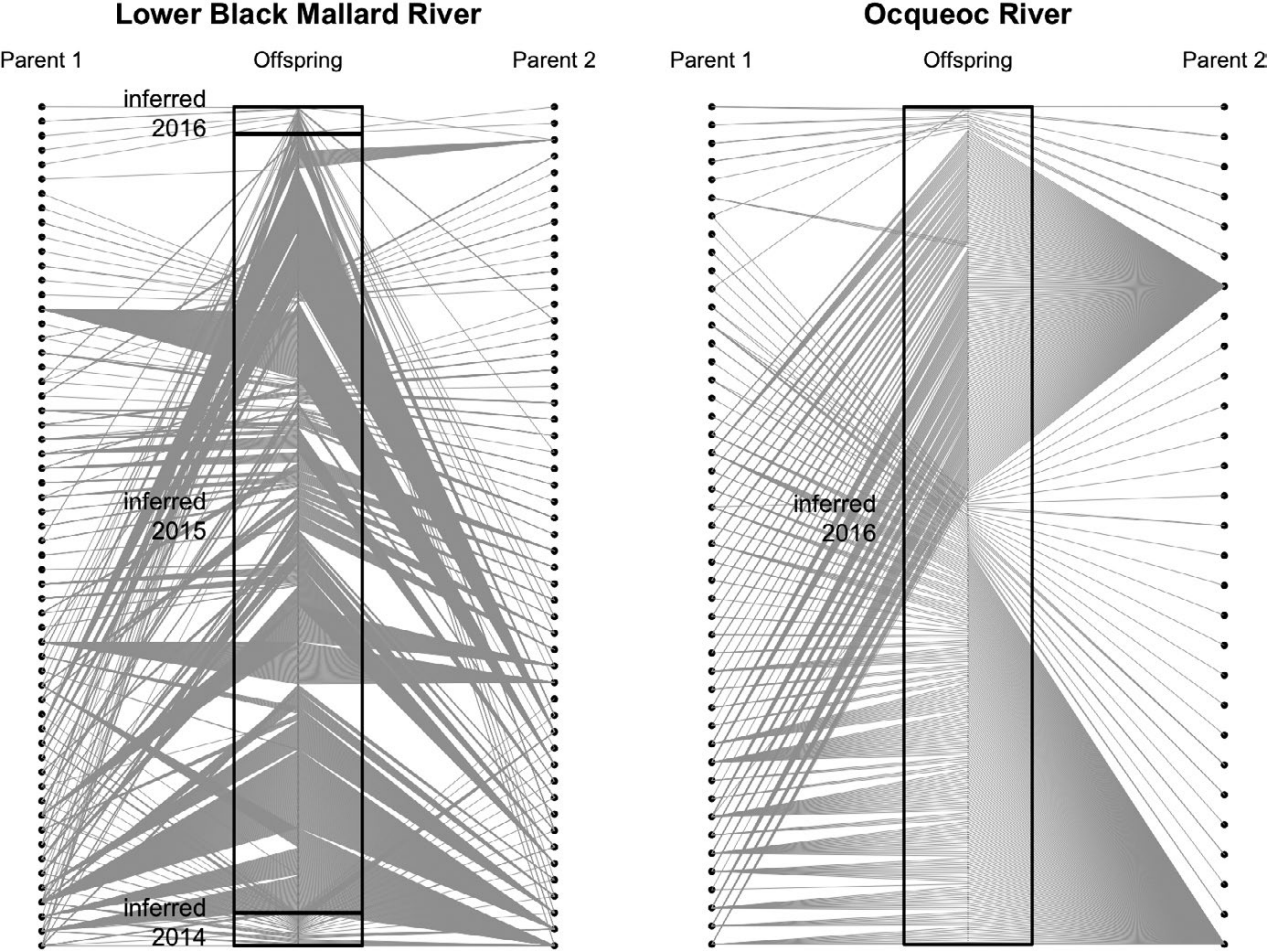
Visualization of reconstructed sea lamprey pedigrees. The center represents genotyped individuals, and dots represent inferred parents. Lines connect each reconstructed parent to sequenced offspring in the pedigree. Black boxes represent cohorts inferred by the mixture method. Note: Since parents were not sequenced, and due to the lack of known sex‐determining genes for sea lamprey, the sex of reconstructed parents cannot be determined. Parent 1 and Parent 2 are used instead

In the Lower Black Mallard River, two age classes were identified based on cluster‐determining methods for both collection years, shown in the histograms in Figure 3. The number of cohorts was determined by consensus for the 2018 collection, and for the 2017 collection, the Rousseau and Mengersen (2011) criteria supported a model with *K* = 2 with higher posterior probability (Table 1). Length distributions among the inferred age classes overlapped, with the exception of a small group in the Lower Black Mallard River 2017 collection, as shown by the boxplots in Figure 4. The reconstructed pedigree for Lower Black Mallard samples had 104 full‐sibling families and 14 Colony clusters. Figure 5 visualizes the family structure across both collections compared to the inferred cohorts from the mixture analysis.

**TABLE 1:** Summary of results for identifying the optimal number of clusters (K) in the mixture analysis for sea lamprey

K	R&M Criteria	BD‐MCMC
Lower Black Mallard River—2017 Collection (n = 386)		
1	0.074	0.008
2	**0.912**	0.067
3	0.013	0.385
4	0.000	**0.540**
Lower Black Mallard River—2018 Collection (n = 614)		
1	0.008	0.112
2	**0.827**	**0.478**
3	0.164	0.319
4	0.000	0.091
Ocqueoc River—2018 Collection (n = 396)		
1	**0.998**	0.143
2	0.002	**0.538**
3	0.000	0.277
4	0.000	0.042

Note

Analyses were performed for each collection and with a range of 1–4 clusters. R&M criteria and Bmixture show the estimated probability of each K value from the Rousseau and Mengersen (2011) criteria and BD‐MCMC, respectively. The highest probability for the number of clusters from each method is bolded.

The largest Colony cluster contained 755 (72%) of the sampled offspring across all collections. The individuals in this cluster were present in both length‐inferred age classes for the 2018 collection and the larger age class in the 2017 collection. The lengths in the largest cluster span across all inferred cohort cutoffs based on the mixture analysis, indicating that the mixture analysis is oversplitting the 2018 collection. No sibling relationships were inferred between the Lower and Upper Black Mallard River collections, indicating that larvae in these two areas were produced by different sets of spawning adults.

Across all three Black Mallard collections, there were 18 Colony clusters of larvae in the full pedigree. Four Colony clusters represent the Upper Black Mallard collection and are not present in the other two collections. In the 2017 collection, there are three Colony clusters exclusively characterized by larval lengths less than 50mm (clusters 2,3, and 13). Larvae identified to be associated with other Colony clusters had median lengths of approximately 75mm. In the 2018 collection, all 14 clusters were associated with individuals with median lengths of greater than 75mm, but less than 100mm (Figure 4). The Colony clusters with a median length of less than 50mm in the 2017 collection were sorted into a separate cohort by the mixture analysis. Based on criteria outlined above and in Figure 2, cluster 2,3, and 13 were determined to be a separate cohort (listed as 2016 in Table 2), and the other clusters were combined into a different cohort (listed as 2015 in Table 2).

**TABLE 2:** Estimates of the effective number of breeding adults and the number of distinct inferred parental genotypes in the pedigree (*N*_s_) for each stream and sea lamprey cohort

Location	Full‐sibs	Clusters	Cohort	n	$\bar{k}$	*V*_k_	LD	SF	*N*_s_	${\hat{N}}_{s}$‐ Chao	${\hat{N}}_{s}$‐ Jackknife
Lower Black Mallard River (A)	96	11	2015	1011	20.22	918.39	24 (22–25)	31 (20–52)	100	122 ± 13	120 ± 5
Lower Black Mallard River (A)	8	3	2016	29	4.14	26.55	3 (2–3)	6 (3–20)	14	45 ± 28	22 ± 4
Upper Black Mallard River (A)	9	4		34	5.23	24.02	3 (2–6)	7 (4–21)	13	15 ± 3	16 ± 2
Ocqueoc River (B)	87	17		389	10.24	799.50	50 (46–55)	9 (5–24)	76	91 ± 8	99 ± 6
Pigeon River (C)	6	3		19	3.17	4.81	8 (3–22)	10 (5–28)	12	16 ± 5	16 ± 3

Note

Locations are shown with the letter abbreviations from the table in Figure 1. Full‐sibs and Clusters refer to the number of full‐sibling groups and Colony cluster groups in the reconstructed pedigree for each stream population, and the cohort is the inferred spawning year for locations with multiple cohorts. n is the number of larval sea lamprey for each cohort inferred by combining Gaussian mixture analysis and reconstructed pedigree data. *V*_k_ and $\bar{k}$ represent the variance in reproductive success and mean number of offspring for contributing parents in the represented stream population, respectively. LD refers to *N*_b_ estimates from the linkage disequilibrium method and SF refers to *N*_b_ estimates from the sibship frequency method. ${\hat{N}}_{s}$ – Chao and – Jackknife represent ${\hat{N}}_{s}$ estimates using the Chao and the jackknife methods, respectively

The mixture models for the Ocqueoc River indicated that one age class of individuals had been collected (Table 1, Figure 3). The pedigree reconstruction contained 17 clusters and 87 full‐sibling families. The pedigree reconstruction contained two half‐sibling families that contributed 91% of sampled offspring (Figure 5). All the individuals from those families were collapsed into the same Colony cluster (Figure 4).

Cluster probability (the probability that a Colony cluster cannot be split) was inconsistent for pedigrees derived from the Ocqueoc and the Lower Black Mallard Rivers. The cluster probabilities for the largest cluster in both systems was <0.5, while small clusters in each location had higher probability (Figure 4). As Colony clusters get larger, probabilities tend to decrease due to compounding uncertainty from each individual relationship in the pedigree. The above analysis was repeated using full‐sibling groups rather than Colony clusters to quantify the differences that could have occurred from low‐probability clusters, and no differences were found.

The reconstructed pedigree in the Pigeon River had six small full‐sibling families that were mostly unrelated to each other. The sample size from the Pigeon River was too small to quantitatively compare inferred cohorts and the family structure from the reconstructed pedigree or run mixture models.

### 3.3 *N*_b_ and *N*_s_ calculations

**FIGURE 6:**
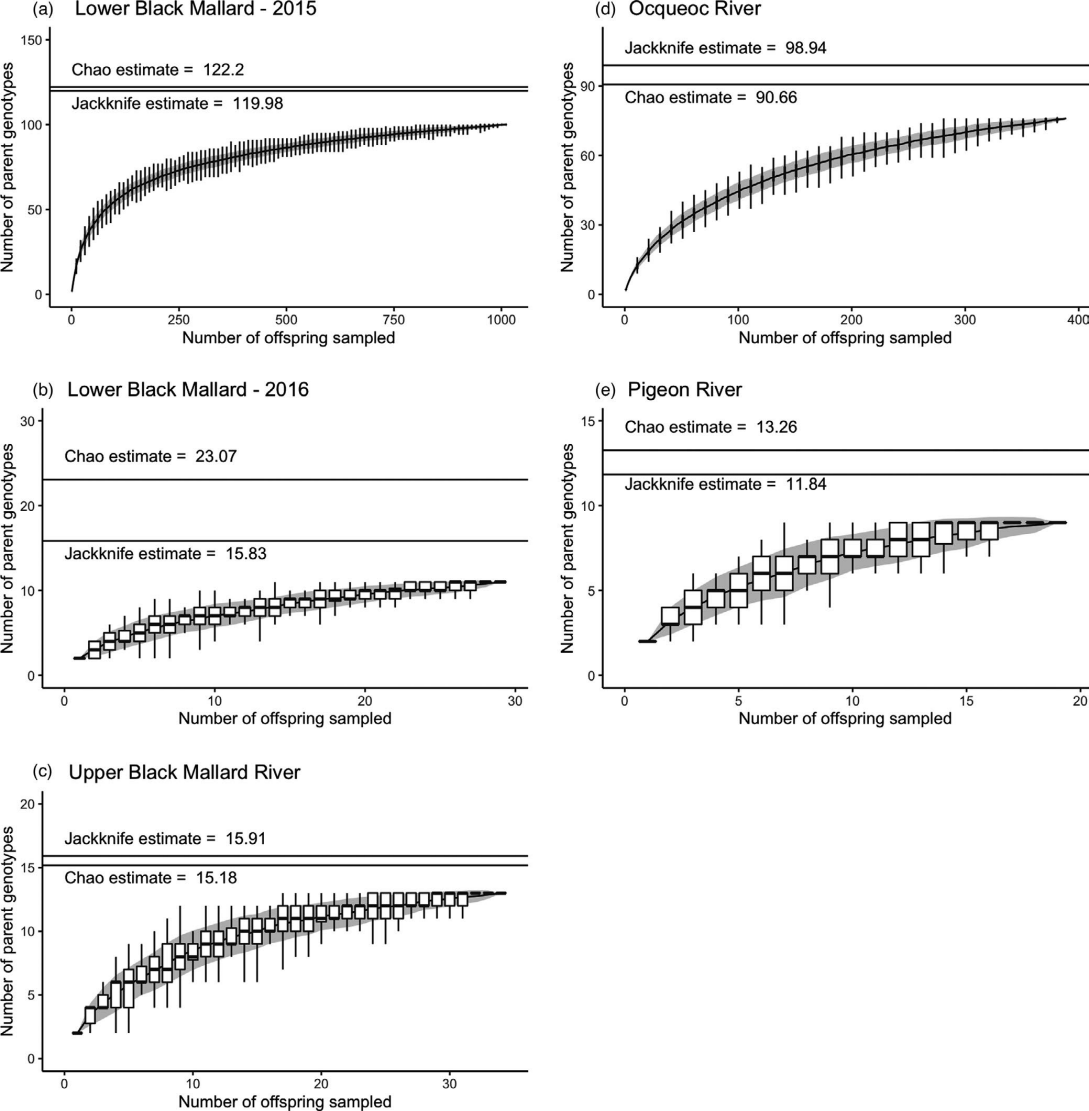
The estimated number of different parental genotypes in the pedigree (${\hat{N}}_{s}$) characterized using pedigree accumulation curves for all three stream systems. For all locations, boxplot distributions for each step size overlay a line plot with a gray background for +/‐ one standard error, and labeled horizontal lines represent ${\hat{N}}_{s}$ estimates from the jackknife and chao methods. Due to the large number of individuals, the Ocqueoc River boxplots are plotted in step sizes of 5 sampled individuals and the Lower Black Mallard River boxplots are shown for sample sizes increasing by 10 individuals. The boxplots for all other locations are plotted for a step size of 1 sampled individual

*N*_b_ and *N*_s_ estimates for all cohorts are summarized in Table 2, and ${\hat{N}}_{s}$ accumulation curves are shown in Figure 6. For the Lower Black Mallard River, the *N*_b_ estimates for the 2015 cohort ranged from 24 to 31 (Table 2) and accumulated *N*_s_ ranged from 120 to 122 (Table 2). The 2016 cohort had *N*_b_ estimates that ranged from 3 to 6 (Table 2) and *N*_s_ estimates that ranged from 22 to 45 (Table 2, Figure 6). For the Upper Black Mallard River collection, *N*_b_ estimates ranged from 3 to 7 (Table 2) and *N*_s_ estimates ranged from 15 to 16 (Table 2, Figure 6). In the Ocqueoc River, *N*_b_ estimates ranged from 9 to 50 (Table 2) and *N*_s_ estimates ranged from 91 to 99 (Table 2, Figure 6). Confidence intervals were small, partially due to the large numbers of loci used in the estimates. *N*_b_ estimates for the Pigeon River collections ranged from 8 to 10 (Table 2), while Chao and jackknife estimates of *N*_s_ were both 16 (Table 2, Figure 6).

## 4 DISCUSSION

In three systems with potential barrier failures implied by the presence of larval sea lamprey populations, *N*_b_ and *N*_s_ were successfully estimated for stream cohorts to assess the effectiveness of barriers. *N*_b_ and *N*_s_, along with reconstructed pedigree data, indicated that in the Black Mallard and the Ocqueoc River, systemic barrier failure was unlikely despite the presence of larvae. Additionally, reconstructed pedigrees were used to correct age classification from length‐based mixture models. These age‐specific cohorts were produced by a relatively small group of successful spawning adults, as indicated by *N*_b_ and *N*_s_ estimates, implying that even a minor barrier breach can lead to a significant larval population in a stream. Overall, larval sequences provided additional information on sea lamprey spawning adult populations that were used to evaluate control measures implemented by management agencies.

### 4.1 *N*_b_ and ${\hat{N}}_{s}$ estimates

Genetic estimates of *N*_b_ and ${\hat{N}}_{s}$ allow inferences pertaining to the number of successful spawning adults contributing to individual cohorts. *N*_b_ and ${\hat{N}}_{s}$ both provide information about spawning populations that can be used to make inferences in management and conservation contexts. In the sampled systems, *N*_b_ estimates and reconstructed pedigrees indicated skewed sex ratios in the Ocqueoc River. ${\hat{N}}_{s}$ provided insight into the number of successfully spawning adults upstream of control barriers. Despite the small to moderate effective breeding sizes estimated for each sampled cohort, larvae were abundant in all systems (estimates range from approximately 3500 larvae in the Upper Black Mallard River in 2017 to 124,000 larvae in the Pigeon River in 2019; unpublished data, A. Jubar, USFWS). In all systems, the vast majority of individuals had half‐ and full‐sibling within the areas sampled. In the Ocqueoc, 91% of individuals were in two half‐sibling families. In the Black Mallard River, 72% of individuals were in a single Colony cluster, and over 97% of the individuals were determined to be in a single cohort from 2015, prior to the barrier construction. Results demonstrate that for species like sea lamprey with high reproductive potential, cohort recruitment levels can be high even in situations where few spawning adults are present.

Increasing sample size and the number of loci analyzed improves *N*_b_ estimates for both methods (England et al., 2006; Wang, 2016; Waples, 2016). Based on simulations conducted by Sard et al. (2020), a high degree of accuracy in the pedigree assignments from Colony is expected given the expected spawning adult population size for these systems and the number of SNP loci used for the analysis. The large number of SNP loci used for pedigree reconstruction and *N*_b_ estimation resulted in high confidence in inferred relationships and confidence intervals that were substantially smaller than those for typical microsatellite datasets (Flanagan & Jones, 2019; Robinson & Moyer, 2013). For the LD estimates, confidence intervals can be artificially narrowed by large numbers of loci, although the corrected jackknife confidence interval approach reduces this effect (Waples et al., 2021). Additionally, the high cluster probabilities for large Colony clusters in the Black Mallard and Ocqueoc Rivers bolster confidence in the family relationships identified by Colony. However, individual misassignment could stem from several potential sources. Pedigree reconstructions for the Black Mallard and Ocqueoc Rivers also contain a small group of individuals that were unrelated to any large family groups. These outlier groups are most likely unrelated individuals, but they could be the result of Colony assignment error (Butler et al., 2004). Outlier groups were confirmed to be sea lamprey based on comparisons with native lamprey (*Lethenteron appendix*, *Ichthyomyzon fossor*), so species misidentifications are considered unlikely in this case. Additionally, there are some differences between the LD method and the SF method of estimating *N*_b_. In the Ocqueoc, the LD estimate was higher than the SF estimate, and in the 2016 cohort of the Black Mallard, the LD estimate is lower. This could be due to differences in assumptions and effects on the estimates between methods, or misassignment of individuals to kin groups that could also have affected mean ($\bar{k}$) and variance (*V_k_*) of adult reproductive success (number of offspring assigned based on the pedigree produced from the full‐likelihood implementation in Colony).

Our results provide an empirical application of ${\hat{N}}_{s}$, a comparatively new method of quantifying spawning adults. Previous work has used accumulation curves to evaluate spawner abundance in green sturgeon (*Acipenser medirostris*; Israel & May, 2010) and Chinook salmon (*Oncorhynchus tshawytscha*; Rawding et al., 2014). ${\hat{N}}_{s}$ has been used for lake sturgeon (*Acipenser fulvescens*) previously to estimate the number of adults recruited to a spawning site (Hunter, 2018; Sard et al., 2021). Given sufficient sample sizes, this method can be used to estimate the number of adults contributing to a cohort (Figure 6). *N*_s_ estimates without an accumulation method have direct dependence on sample size since they are calculated as the number of distinct reconstructed parental genotypes for a set of offspring and are thus limited by sample size. By applying methods designed to estimate total species richness to reconstructed pedigrees, that dependence is reduced.

### 4.2 Cohort identification

Mixture analysis in sea lamprey has several sources of uncertainty. Techniques rely on the presence of several large cohorts in a stream sample to provide accurate cohort assignments and are expected to be most effective for age‐0 and age‐1 individuals where length distributions are more distinct from older cohorts (Dawson et al., 2009). Additionally, environmental conditions affect the growth rate of larvae. Variables such as growing degree days, stream temperature, and larval sea lamprey density are all significant predictors of larval growth in streams (Dawson et al., 2021).

*N*_b_ and *N*_s_ are both estimates generated for a single spawning year, meaning that the ability to separate offspring into cohorts is vital for accurate estimates. Combining Gaussian mixture models with reconstructed pedigree data allows for the identification of potentially misidentified cohorts from the length data alone, minimizing error in cohort identification. Including individuals from multiple cohorts in *N*_b_ and *N*_s_ calculations generated from the reconstructed pedigree would upwardly bias estimates due to the inclusion of parents from multiple spawning events (Wang et al., 2016). For the linkage disequilibrium estimates, mixture LD, linkage that arises from pooling two separate spawning groups, would lead to a downward bias (Waples & England, 2011).

Uncertainty in the cohort assignments from the mixture analysis was evident in the Lower Black Mallard River samples. Larvae were separated into multiple cohorts with overlap between length distributions for individuals assigned to older cohorts. Additionally, variability in growth within age classes was greater than previously assumed (Figure 4), potentially contributing to the oversplitting of larval cohorts observed in both streams. Incorporating family pedigree information further supported the conclusion that the number of cohorts was overestimated by the mixture analysis, as several sibling groups spanned multiple inferred cohorts. For both collections in the Lower Black Mallard River, length‐based mixture analysis divided members of the largest family cluster into two cohorts, again indicating oversplitting. In semelparous species like the sea lamprey, family structure present in reconstructed pedigrees can be combined with length data as complementary information to verify cohort assignments. The addition of a check on the mixture analysis using family structure allows for the identification of misassigned individuals or oversplit cohorts that could not be identified using length data alone to age individuals.

### 4.3 Application of results

Population estimates across all three streams sampled in this study imply that barriers limited adult spawning numbers but were not completely effective at blocking access to spawning habitats. Thus, the large numbers of larvae present in sampled systems were a poor indicator of spawning adult abundance, which is an important finding for managers. Another important finding was that members of full‐ and half‐sibling families were identified in multiple year cohorts, which is impossible due to the species’ semelparous life history. Cohort assignments identified by mixture models (i.e., in the absence of confirmatory genetic data) showed that length‐based analysis alone does not provide accurate cohort assignments. Our analyses illustrate the potential to improve cohort assignments by incorporating population genomic data and pedigree analysis for sampled sea lamprey larvae. Collectively, effective size, minimum spawning size estimates, and reconstructed pedigrees based on larval sequencing were successfully used to make inferences about spawning adult populations in three streams.

Population genomic data were used to infer aspects of sea lamprey biology that contribute valuable information for sea lamprey assessment. Results from the Lower Black Mallard River indicated that the majority of individuals originated from a single cohort due to the existence of full‐sibling relationships between inferred cohorts from the mixture analysis. These data are consistent with the expectation that a moderate number of adult sea lamprey spawned in the Black Mallard River in 2015 after lampricide treatment, but prior to the electric barrier installation in 2016. Collectively, our data suggest that the electric barrier in the Black Mallard River was effective at reducing sea lamprey migration upstream, as *N*_b_ of the 2016 cohort was much smaller than *N*_b_ of the 2015 cohort, and a 2017 cohort was not confidently identified by our mixture analyses for the Lower Black Mallard River collections. There are alternative explanations for small *N*_b_, such as high variance in reproductive success and strongly skewed sex ratios, as seen in the Ocqueoc River estimates. Additionally, the lack of family relationships between the Upper and Lower Black Mallard River implies two separate subsets of spawning adults. In the Ocqueoc River, 91% of larvae was from two half‐sibling families, indicating that a small group of fertile males were present above the barrier along with the females released for research experiments. Estimates from samples collected in the Pigeon River indicated that both *N*_b_ and *N*_s_ were small, which is consistent with the expectation that releases of sterile males decreased the number of successful spawning adults in the system.

Although sea lamprey are invasive in the Great Lakes, they are endangered in parts of Europe, and conservation efforts are underway to protect declining populations (Hansen et al., 2016). Many of the same questions related to management of invasive Great Lakes populations also apply to threatened marine sea lamprey populations spawning in North American and European tributaries of the Atlantic Ocean. Estimates of *N*_b_ and the per‐generation effective population size (*N_e_*) can provide important information on patterns of relatedness, the rate of diversity loss due to genetic drift and inbreeding, and the species’ potential for adaptation.

Population genomic data, including estimates of effective size, have been used as a monitoring tool in many conservation and management situations for other species, such as translocations and reintroductions (Hess et al., 2015; Roques et al., 2018; Whitlock et al., 2017), quantifying genetic diversity to prevent extinctions (Faulks et al., 2017), and identifying ecologically significant units (Blower et al., 2012). Parentage has been used to evaluate the size of invading populations in species like the Asian swamp eel (*Monopterus albus*; Taylor et al., 2021). Genetic data were used in all of the above situations to evaluate the population or assess the success of a management action, and this type of assessment is increasingly needed among managed populations (Hoban et al., 2021). Thus, population genomic data and estimation of effective population sizes could be used to assess the efficacy and level of success of management actions related to invasive species, endangered populations, species of conservation concern, and managed species (Nunziata & Weisrock, 2018). Recent developments, including the availability of a reference genome (Smith et al., 2013, 2018) and the RAD‐Capture marker panel (Sard et al., 2020) employed in this study, position Great Lakes sea lamprey as an emerging model system for the study of species invasions.

## CONFLICT OF INTEREST

The authors declare no conflict of interest.

## Supporting information

Fig S1

## Data Availability

All genomic reads generated for this project have been uploaded to NCBI Short Read Archive (Accession Number: PRJNA763927). Length data for all individuals are available on GitHub along with all scripts generated for the project (https://github.com/weiseell/NbdLamprey).
