# Supplementary material for: Pedigree analysis and estimates of effective breeding size characterize sea lamprey reproductive biology
Source: Evol Appl. 2022 Mar 15;15(3):484–500. doi: 10.1111/eva.13364 (PMC8965388; doi:10.1111/eva.13364)

Supplmental Figure 1A: Black Mallard River

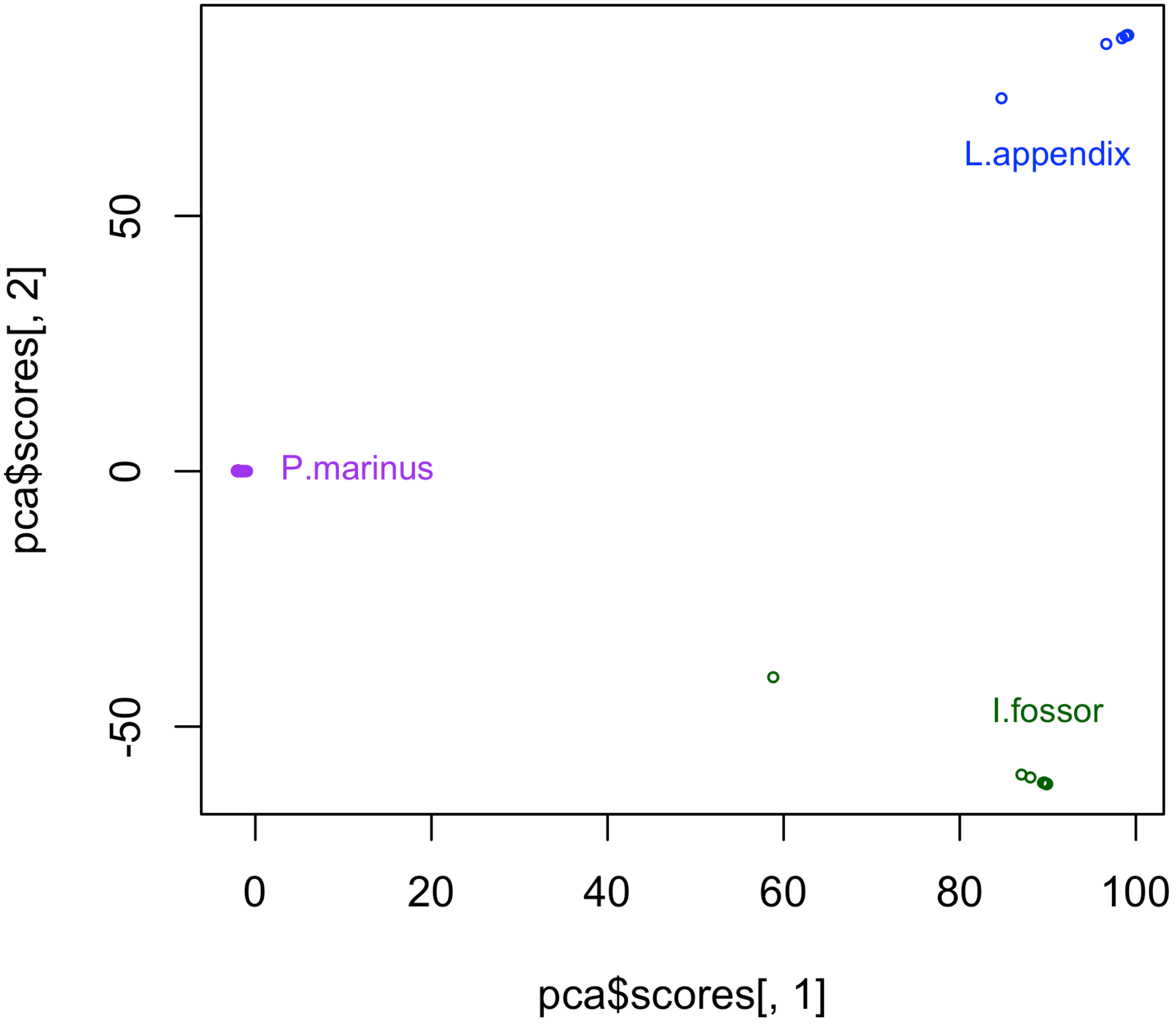

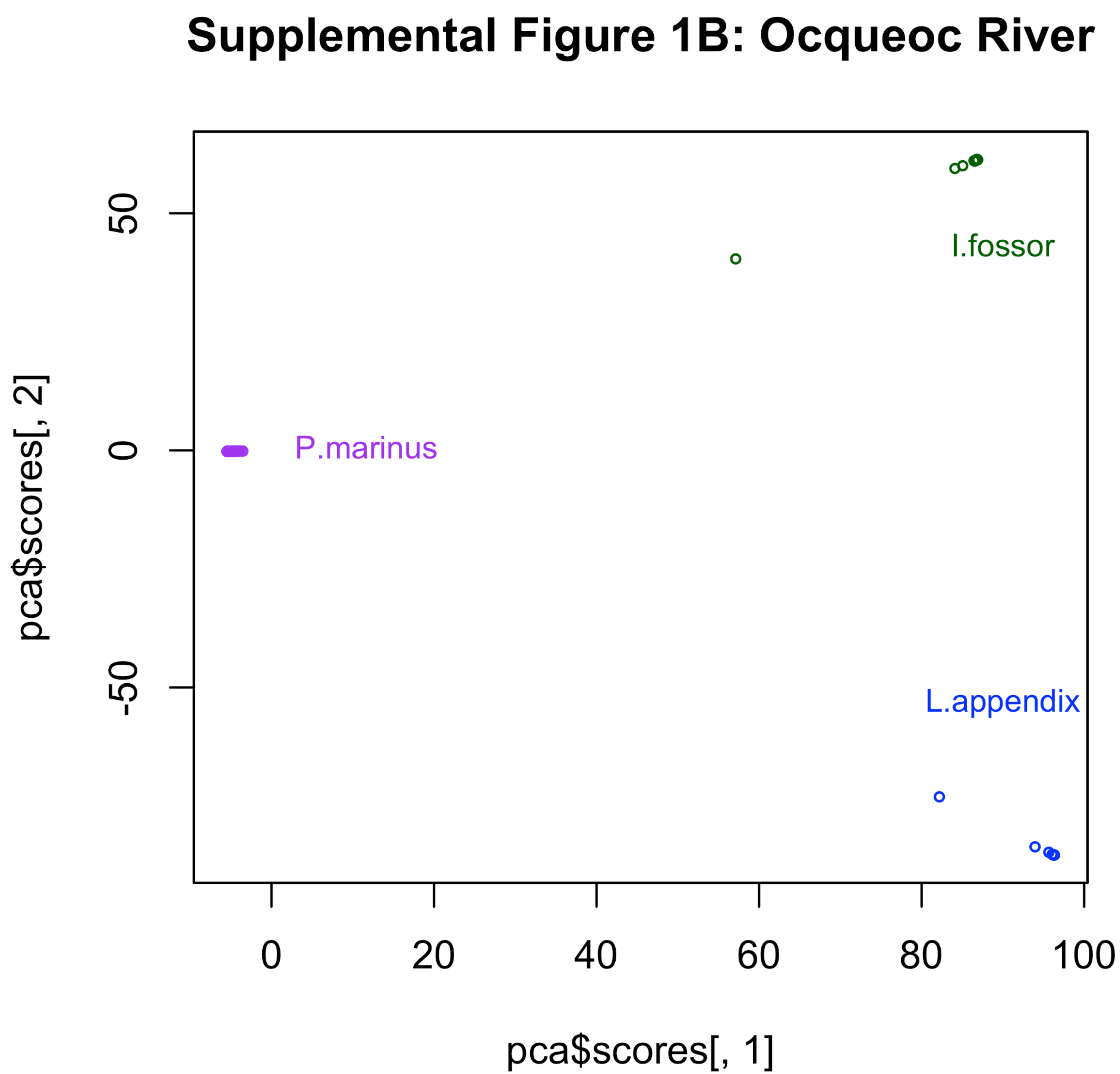
Supplemental Figure 1B: Ocqueoc River
I.fossor
P.marinus
L.appendix
50
0
-50
0
20
40
60
80
100
pca$scores[, 2]
pca$scores[, 1]

Supplemental Figure 1C: Pigeon River

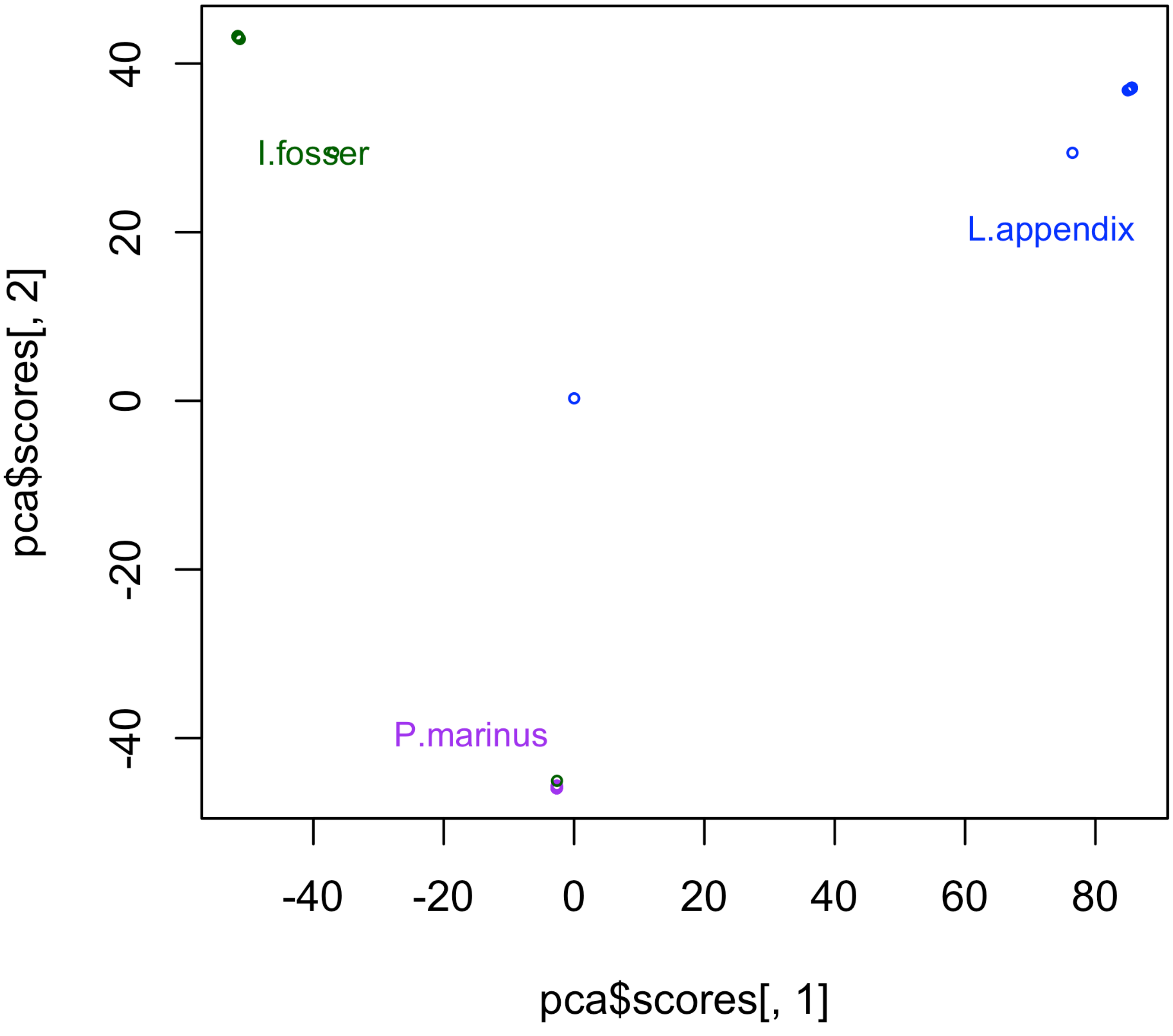

Supplement: Supplementary file 1 — Fig S1 [file EVA-15-484-s001.pdf]
